# Domain-informed density extraction for robust mental health classification of long social media posts

**DOI:** 10.3389/frai.2026.1861620

**Published:** 2026-08-27

**Authors:** Rafat Hammad, Suha Rababah

**Affiliations:** Faculty of Information Technology and Computer Sciences, Yarmouk University, Irbid, Jordan

**Keywords:** data leakage, deep learning models, long-document classification, machine learning, mental health classification, natural language processing, text segmentation

## Abstract

**Background:**

Recent advances in machine learning and natural language processing (NLP) have enabled the early identification of mental disorders from social media content. Among such platforms, Reddit is characterized by linguistically rich, long-form narratives in which users describe their psychological symptoms, emotions, and personal experiences. However, these datasets present significant challenges for text classification because the posts are lengthy, voluminous, and class-imbalanced. In this study, we revisit the concept of a segmentation-based, expression-weighted representation that emphasizes clinically relevant language while suppressing irrelevant content. During this investigation, we identify a critical limitation in conventional preprocessing pipelines—specifically, the application of chunking and oversampling prior to the train–test split—which introduces data leakage and consequently inflates reported performance. To address this issue, we propose a leakage-safe evaluation protocol together with a domain-informed density extraction method that identifies clinically dense passages using a lexicon derived exclusively from the training data.

**Results:**

The proposed method was evaluated using logistic regression, linear SVM, XGBoost, fastText, RNN, and TextCNN, together with a transformer-based baseline (MentalBERT), all under a leakage-safe evaluation protocol. After eliminating data leakage, the apparent advantage of naive weighted chunking disappeared, and the headline macro-F1 score decreased from approximately 0.96 to 0.67. In contrast, the proposed domain-informed density extraction method consistently outperformed fixed-window chunking in five of the six models and matched or exceeded the full-text baseline in several cases. Statistically significant improvements were observed for logistic regression, RNN, and fastText, despite using only a fraction of the input text. For the context-limited transformer, density-based selection significantly outperformed naive truncation (macro-F1 + 0.023, 95% CI [+0.006, +0.041], *p* = 0.008).

**Conclusion:**

Our findings demonstrate that a leakage-safe evaluation protocol is essential for producing credible results in mental health classification from long social media posts. They further show that domain-informed density extraction provides a robust and practical text representation, with its greatest benefits emerging when models are unable to process the entire post. By selectively preserving clinically informative content while reducing input length, the proposed approach offers an effective and computationally efficient alternative to increasingly complex model architectures.

## 1 Introduction

### 1.1 Background

Social media has become an indispensable source of information on mental health since it allows people to speak their minds to a larger audience than they normally would in person. This kind of data can help diagnose mental disorders in addition to the information gathered during a consultation with the psychiatrist because these platforms might provide implicit signals that can help diagnose and intervene in mental health problems earlier (Calvo et al., 2017). Natural Language Processing (NLP) allows computers to comprehend human language and analyze its syntax, semantics, and sentiment using computational and statistical methods. Modern NLP algorithms, such as recurrent neural networks (RNNs), long short-term memory networks (LSTMs), and the BERT model, are efficient in capturing the context and emotions of people, so they can easily be used in text classification and prediction tasks (Tanana et al., 2021).

The gathering of data is the basis of machine learning and NLP applications in medicine, and there are different ways to collect the necessary data from social media platforms, patients’ reviews, and clinical data (Parrott et al., 2020; Herrera-Peco et al., 2023; Zhang et al., 2022). Social media text, for example, could be an invaluable source of information on people who are currently going through psychological issues and should be referred to professional help as soon as possible. This is why sentiment analysis of social media texts has become a common method in the detection and assessment of depression (Babu and Kanaga, 2022). On the other hand, capturing human emotions via computational methods becomes difficult due to the diversity of languages, context, culture, and even the quantity and quality of the social media datasets could influence the model (Nijhawan et al., 2022; Wang et al., 2020). Nevertheless, advances in machine learning and NLP have reached a stage where they can contribute meaningfully to healthcare by providing data-driven insights that complement, rather than rely solely on, individual clinical judgments (Forsting, 2017; Farsi, 2021; Ventola, 2014).

### 1.2 Aim of the research

The timely and accurate identification of mental health signals is essential for improving diagnosis and treatment outcomes, thereby mitigating the impact of mental disorders. Although NLP models can extract valuable information from text, their effectiveness depends heavily on the nature and organization of the training data and—as demonstrated in this study—on the integrity of the evaluation protocol used to assess them. This work aims to achieve a more context-aware interpretation of mental health-related text by training language models on long Reddit posts under a methodologically rigorous and leakage-safe evaluation framework. The main contributions of this study are summarized as follows:

*Leakage-safe evaluation protocol*: We demonstrate that the common practice of applying chunking and oversampling to long posts before the train–test split can introduce data leakage, as chunks originating from the same post, as well as duplicated oversampled instances, may appear in both the training and test partitions. To address this issue, we introduce a leakage-safe evaluation protocol in which the train–test split is performed at the post level before any chunking takes place, oversampling is applied exclusively to the training partition, and all performance metrics are reported at the post level together with bootstrap confidence intervals.

*Domain-informed density extraction*: Rather than constructing domain-specific regular expressions manually, we suggest a density-based approach that is capable of extracting multi-regional information based on a discriminatory lexicon that is extracted purely from the training dataset via a log-odds estimator utilizing an informative Dirichlet prior. In order to avoid any possibility of label leakage, disorder names are omitted from the lexicon, guaranteeing that the extraction is based on symptom-related lexicon and not on explicit class labels. Moreover, the lexicon is used in all models, which makes the resulting representation reproducible and model-agnostic.

*Rigorous, statistically grounded benchmarking*: We revisit six classical and deep learning models as well as a transformer-based benchmark (MentalBERT) under the proposed leakage-safe evaluation protocol. The confidence intervals are presented in addition to *p*-values of the corresponding paired *t*-test for transformer comparison. This allows us to evaluate clearly under what circumstances the suggested approach brings benefits and under which it does not, thus eliminating misleadingly high results obtained with the standard pipeline.

The rest of this paper is structured as follows. Section 2 covers the related work. Section 3 introduces the proposed approach and the leakage-free evaluation protocol. Section 4 gives the experimental results, while the discussion is given in Section 5.

## 2 Related work

Studies in the domain of computational mental health have found that language features in the posts from social media sites may function as indicators for mental health conditions, with pronoun use, emotionality, and stylistic aspects being among the useful features for mental state detection. For instance, Jiang et al. (2020) constructed a dataset of Reddit users suffering from various mental illnesses, including depression, anxiety, and bipolar disorder, and proved the usefulness of language features in discrimination between different disorders. Yet, some authors argue that prediction based on language features is extremely difficult because most of them are common to multiple disorders, not specific (Joulin et al., 2017).

Classical machine learning algorithms such as logistic regression, SVM, random forests, and gradient boosting decision trees are still popular due to their simplicity, interpretability, and computational efficiency. Zhang et al. (2025) introduced the universal scheme combining TF-IDF features, word embeddings, and sentiment lexicons along with proper data splitting, cross-validation, and measures of model quality such as F1 score and AUPRC for imbalanced mental health datasets. Gradient-boosting methods, in particular, are well-suited to capturing nonlinear relationships in sparse textual data and therefore provide a strong baseline for text classification tasks.

With the growing prevalence of mental health discussions on social media, deep learning approaches have become increasingly dominant. Convolutional neural networks (CNNs), long short-term memory networks (LSTMs), and transformer-based models have consistently outperformed traditional machine learning approaches on tasks involving emotions and mental health conditions such as depression. Saeed and Ahmed (2025) proposed a BiLSTM framework for Reddit that combined textual content with temporal posting information, achieving an accuracy of 74.55% and an F1-score of 0.7376. Widjayanto and Setiawan (2025) reported accuracies of up to 84.67% on Indonesian tweets using CNN–BiLSTM architectures with BERT and FastText embeddings, while Zhou and Mohd (2025) enhanced BERT–BiLSTM models through emoji normalization, slang substitution, and emotion annotation.

Hybrid models have also been successful. For instance, Alqazzaz et al. (2023) managed to reach the accuracy of 86.2% when detecting depression on Twitter, while Amanat et al. (2022) reached an accuracy of more than 90% in a model combining LSTM, PCA, TF-IDF, and one-hot encoding techniques. When detecting depression on Reddit, Figueiredo et al. (2022) used CNN classifiers with standard word embeddings and reached an accuracy of 76%. In turn, attention mechanisms added to the BiLSTM models proposed by Almars (2022) increased the accuracy up to 83% despite the use of a small dataset, demonstrating how effective attention mechanisms could be even when there is not much data. Multi-platform research has become popular in recent years. Thus, for example, Vasha et al. (2023) report the accuracy of 75.15% on SVMs on merged datasets, while Wani et al. (2022) show that larger and multi-sourced datasets increase the predictive power but also the complexity of the data processing and risks of bias. The BERT–CNN framework was suggested by Gorai and Shaw (2024) for the purpose of assessing suicidality risk on Twitter and Reddit, while Fudholi and Abdurrahim (2024) developed a CNN–BiLSTM model with pre-trained FastText embeddings for several disorders, reaching the accuracy of 85%. Characterization studies of shared tasks on Reddit have helped in identifying suicidal risk factors and specific linguistic signs of disorders (Zirikly et al., 2019; Gkotsis et al., 2017).

Overall, the literature demonstrates a clear progression from conventional machine learning approaches to increasingly sophisticated deep learning architectures for mental health classification from text. It also highlights several factors that influence predictive performance, including linguistic and contextual features, dataset size, preprocessing strategies, and evaluation methodology. A recurring limitation, which this study directly addresses, is the limited attention devoted to evaluation rigor—particularly the interaction between chunking, oversampling, and train–test splitting—as well as the lack of systematic investigation into whether chunk-selection strategies provide genuine benefits for models that are not inherently constrained by input length.

## 3 Methodology

**Figure 1:**
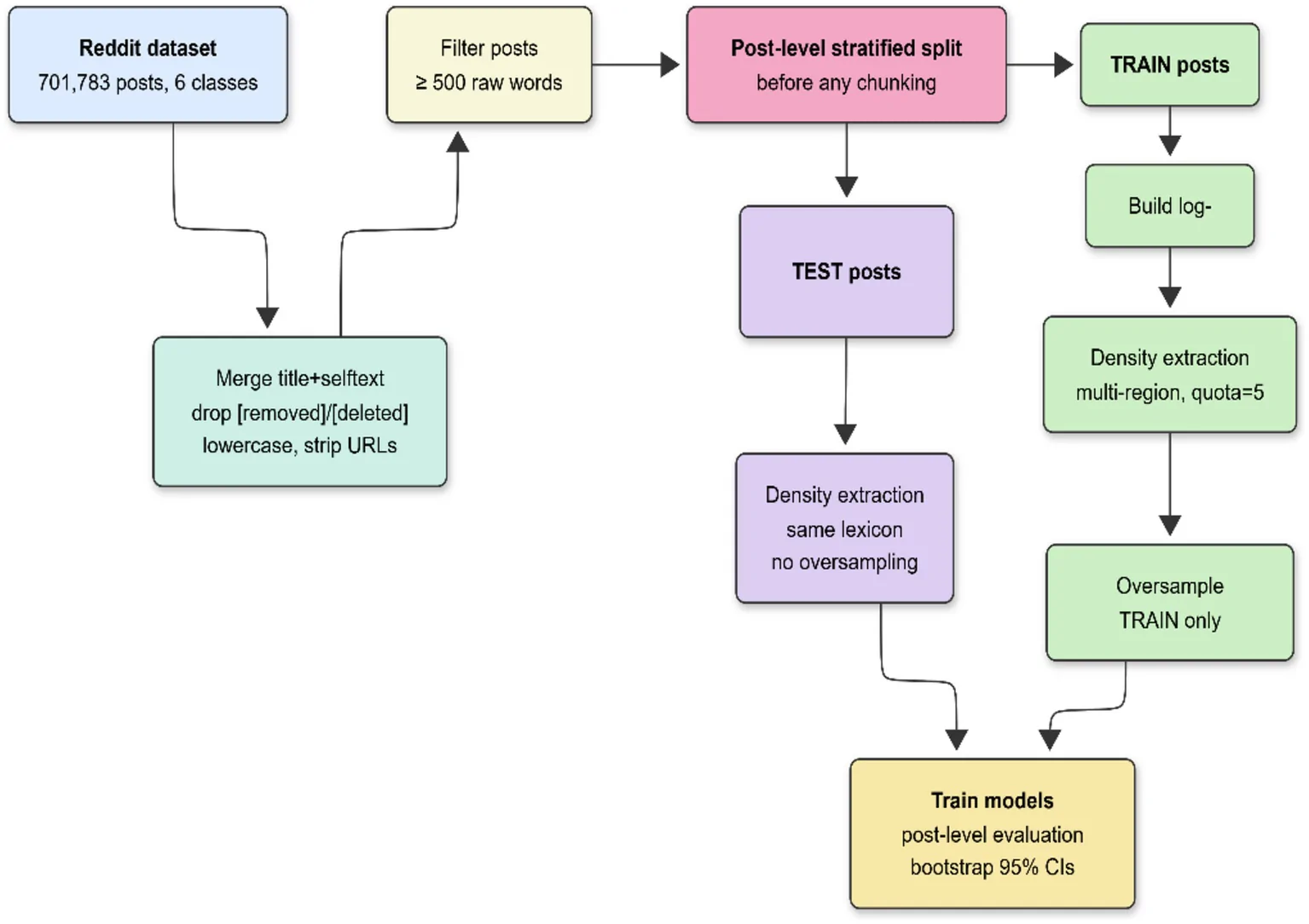
Workflow diagram of the proposed leakage-safe pipeline.

The problem addressed in this work is the development of a pipeline for processing and classifying long Reddit posts to identify indicators of mental health conditions. The proposed pipeline comprises six stages: data acquisition, text preprocessing, a leakage-safe train–test split, construction of a training-derived lexicon with density-based extraction, model training, and model evaluation, as illustrated in Figure 1. To prevent data leakage, the train–test split is performed at the post level before any chunking is applied. The discriminative lexicon is constructed exclusively from the training partition, and oversampling is likewise restricted to the training data. During evaluation, chunk-level predictions are aggregated to produce post-level classifications.

### 3.1 Dataset acquisition

The present work makes use of a publicly available Reddit-based mental health dataset, which includes labeled posts pertaining to various mental disorders, such as anxiety, depression, bipolar disorder, borderline personality disorder (BPD), and schizophrenia (Parrott et al., 2020; Herrera-Peco et al., 2023). The whole dataset is made up of 701,783 posts classified according to six categories of subreddit-based classes. To create a unified textual representation, the title and self-text fields are merged into a single document, combining the summary and elaborative components of each post. Posts that have been removed or deleted are not taken into account in the analysis. The data include posts generated in various years and thus represent a valuable source for studying linguistic patterns related to mental disorders.

### 3.2 Text preprocessing

Text preprocessing is performed in order to minimize noise in the data and unify the textual content. All text is converted to lowercase in order to make the vocabulary less sparse. Regular expressions are used to get rid of URLs and hyperlinks according to common practices of text preprocessing in social media NLP tasks (Sarker and Gonzalez, 2017), while punctuation and English stopwords are removed using NLTK. Removing such elements from the text has been proven to increase the performance of classification models in mental health text analysis (Monroe et al., 2008).

Posts with at least 500 words of raw word count are considered in order to perform chunking. It is assumed that longer posts contain more information about the mental health experiences of people. Such a filtering step allows mitigating possible problems with dealing with long sequences in deep learning models, such as higher memory requirements and vanishing gradients (Pascanu et al., 2013). To maintain a leakage-safe evaluation protocol, each retained post is assigned a stable identifier that is preserved throughout the entire processing pipeline.

### 3.3 Leakage-safe train/test split

The order of operations is the key methodology that has been used in this research work. Under the traditional approach, longer posts are initially split into chunks and oversampled. After that, the chunk-level dataset is further divided into training and testing partitions. As a result, data leakage appears since several chunks, which have originated from the same post, and duplicated chunks formed via the process of oversampling with replacement can be placed in both partitions. Therefore, the model will be validated against the data that it has actually seen during the process of training.

In order to avoid such data leakage, a stratified 80/20 train/test split is conducted at the post level before any chunking takes place. Each of the two partitions is then processed separately, with separate chunking procedures being applied to each of them. The process of oversampling is carried out solely for training data. The integrity of the partitions is ensured by using a unique post ID that is passed along the whole pipeline, and explicit validation that makes sure that there are no overlapping post IDs between the two partitions. Finally, all evaluation metrics are computed at the post level by averaging the results produced by chunks of each post.

### 3.4 Training-derived lexicon and density-based extraction

**Figure 2:**
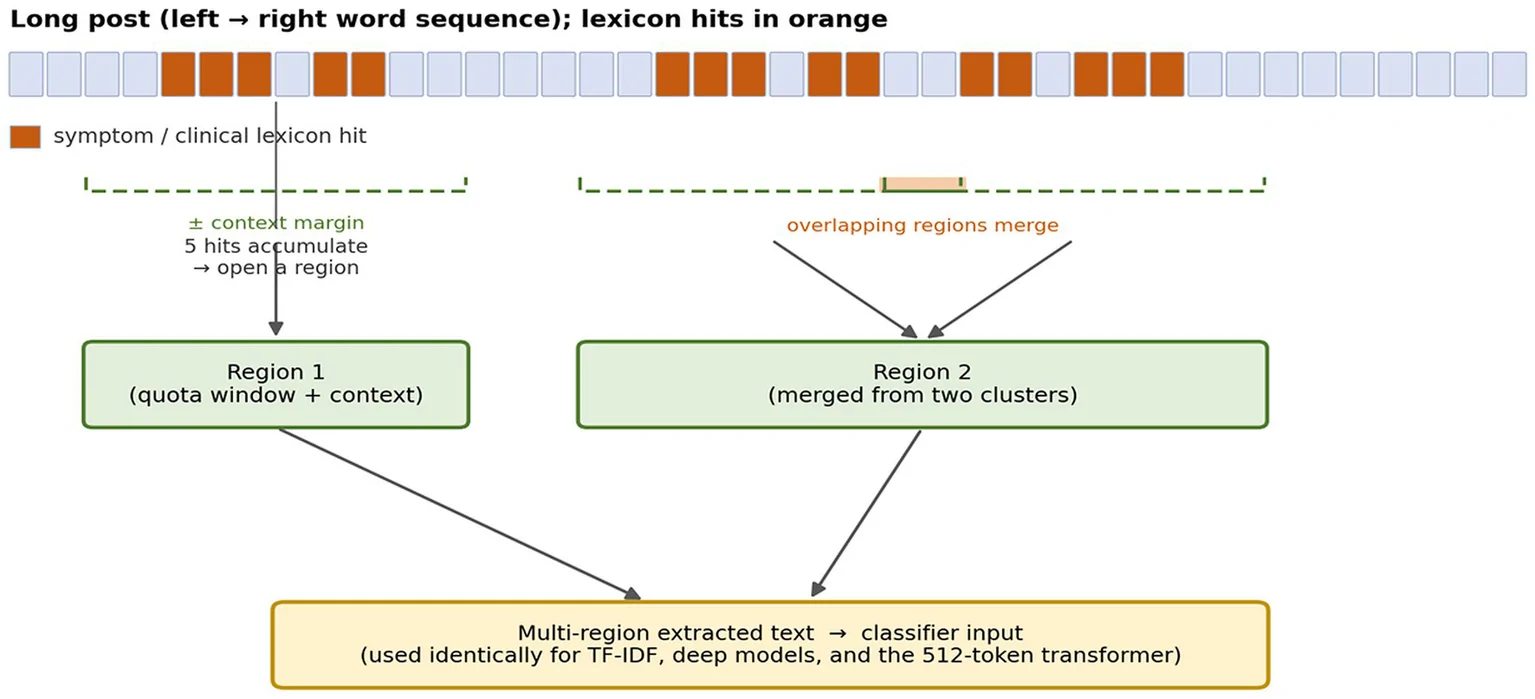
Density-triggered, multi-region extraction.

To assign higher weight to text fragments with clinically useful content, the preceding rule-based weight assignment scheme is replaced by a clear-cut data-driven lexicon created from the training data alone (Figure 2). Each vocabulary word is then weighted according to the logarithm of its odds ratio in relation to the rest of the classes in which it appears, using the Dirichlet prior formulation of Monroe et al. (2008). This approach reduces the influence of rare-word noise and yields more reliable estimates of term discriminativeness. For a term *w* and class *k*, the smoothed log-odds ratio and its corresponding standardized score are defined by Equations 1, 2, respectively:


$$\begin{array}{c} \delta_{w(k)}=\ln[(y_{w(k)}+\alpha_{w})/(n_{k}+\alpha 0-y_{w(k)}-\alpha_{w})]\\ -\ln[(y_{w(-k)}+\alpha_{w})/(n_{-k}+\alpha 0-y_{w(-k)}-\alpha_{w})] \end{array}$$ (1)

$$z_{w(k)}=\delta_{w(k)}/\surd (1/(y_{w(k)}+\alpha_{w})+1/(y_{w(-k)}+\alpha_{w}))$$ (2)

Where *δ_w(k)_*—the log-odds-ratio of term *w* for class *k* versus all other classes (the *¬k* set); *z_w(k)_*—the standardized score; the terms with the highest z per class form that class’s discriminative list; *y_w(k)_, y_w(¬k)_*—the count of term *w* in class *k* and in all remaining classes, computed on the training data only; *n_k_, n_¬k_*—the total token counts in class k and in the remaining classes; *α_w_, α₀*—the informative Dirichlet prior for term w (proportional to its background frequency) and its total mass.

The top 75 terms from each class are selected and combined into a single union lexicon containing 354 unique terms. This design ensures that the extraction process remains label-blind and does not rely on class information during inference. To prevent the lexicon from encoding explicit class labels, disorder and diagnosis names are excluded, ensuring that the selection process is driven by symptom-related and clinically relevant vocabulary rather than the disorder label itself.

Given the token sequence of a post, occurrences of lexicon terms are identified, and their positions are marked. A region is initiated once five lexicon matches have accumulated and is terminated when the gap between consecutive matches exceeds 60 words. Each region is then expanded with a 40-word contextual margin on both sides, and overlapping regions are merged. Consequently, a long post may yield multiple content-centered passages rather than a single contiguous window. Posts that do not contain any qualifying region fall back to a representation based on their leading words. The same extraction procedure is applied uniformly across all model families.

For comparison, two reference representations are also evaluated: the full-text representation (without chunking) and a topic-blind fixed-window baseline consisting of 256-word windows with a 64-word overlap, following established chunking practices in the literature (Johnson and Zhang, 2017; Zhang et al., 2015).

For transparency and reproducibility, Table 1 presents the most discriminative terms identified for each class. Disorder and diagnosis names are excluded to prevent the lexicon from encoding explicit class labels, and a small number of high-frequency colloquial tokens are omitted from the table solely for presentation purposes. These omissions affect only the displayed results and do not alter the lexicon used during extraction.

**Table 1:** Top discriminative terms per class (training-derived log-odds union lexicon; 354 terms total).

Class	Top terms
Anxiety	panic, attack, attacks, heart, doctor, chest, fear, sleep, breathing, blood, symptoms, worry, body, stress
BPD	relationship, hurt, love, together, person, upset, told, relationships, emotions, emotional, boyfriend, abandonment
Bipolar	manic, episode, mania, meds, episodes, lithium, hypomanic, hypomania, lamictal, seroquel, medication, psychotic
Depression	life, school, parents, family, college, die, dad, mom, job, money, anymore, years, live
Schizophrenia	walls, voices, hallucinations, eyes, delusions, psychosis, crawling, psychotic, delusion, exist, run, cars

### 3.5 Model training

Two model families were trained using the extracted text representations: traditional machine learning models and deep learning models. For the traditional machine learning category, Support Vector Machines (SVMs) (Joachims, 1998), logistic regression, and XGBoost (Nalluri et al., 2020) were selected because they remain effective for text classification tasks involving sparse, high-dimensional feature spaces. Textual features for these models were generated using TF-IDF vectorization with a vocabulary size of 100,000 features and the inclusion of both unigrams and bigrams. TF-IDF measures the importance of a term within a document relative to the entire corpus by combining term frequency and inverse document frequency, as defined in Equation 3:


$$w_{t,d}=\mathrm{tf}(t,d)\times \ln(\;N/\mathrm{df}(t))$$ (3)

Where *w_t,d_*—the TF-IDF weight of term t in document (chunk) d; *tf(t, d)*—the frequency of term t in document d; *N*—the total number of documents (chunks) in the training corpus; *df(t)*—the number of documents containing term t.

In addition, we trained several neural architectures that are well-suited to sentence-level and short-sequence text. These included recurrent neural networks (RNNs) for modeling sequential dependencies (Mikolov et al., 2010), TextCNN for capturing local *n*-gram features (Kim, 2014), and fastText, an efficient embedding-based classifier that leverages subword information (Joulin et al., 2017). To provide a transformer-based comparison, we also included MentalBERT (mental/mental-bert-base-uncased) as a baseline model.

Because the dataset exhibits class imbalance, each minority class within the training partition is oversampled with replacement until it reaches the size of the largest class, as specified in Equation 4. To preserve the integrity of the leakage-safe evaluation protocol, this procedure is applied exclusively to the training partition and is never performed on the test set:


$$n_{c}\to n_{\max}=\mathrm{ma}x_{c'}n_{c'}\;\text{for every class}\;c$$ (4)

Where *n_c_*—the number of training chunks in class c before balancing; *n_max_*—the target size after oversampling, equal to the largest class count.

For MentalBERT, which is limited to a maximum input length of 512 tokens, we evaluate two input regimes using the same leakage-safe split: (i) naive head truncation of the raw post and (ii) density-extracted passages. In both cases, inputs are truncated to the model’s maximum sequence length to ensure a fair comparison under identical constraints.

### 3.6 Model evaluation

Models were evaluated on a held-out test set derived from stratified splits. Accuracy measures the proportion of correctly predicted instances, while precision, recall, and F1-score assess class-level performance. To account for class imbalance, the macro-averaged F1-score is reported so that each class contributes equally regardless of its frequency, as defined in Equation 5:


$$F1_{c}=2P_{c}R_{c}/(P_{c}+R_{c}),\;\text{macro}-F1=(1/K)\;\Sigma_{c=1}K\;F1_{c}$$ (5)

Where *P_c_, R_c_*—the precision and recall for class *c*; *F1_c_*—the per-class F1-score; *K*—the number of classes (six in the primary analysis, five after excluding the generic *mentalillness* class).

All reported metrics are accompanied by 95% bootstrap confidence intervals (*B* = 1,000 resamples of the test posts), as defined in Equation 6. For the primary transformer comparison, a paired bootstrap test (10,000 resamples) is additionally performed to assess the difference between the two input regimes on the same test posts. This paired procedure provides a more sensitive and statistically powerful comparison than evaluating overlap between two independent confidence intervals.


$$CI_{95\%}(\theta )=[Q_{2.5}(\overset{⌢}{\theta }(b)),Q_{97.5}(\overset{⌢}{\theta }(b))],\;b=1,\ldots ,B$$ (6)

Where *θ*—the metric of interest (accuracy or macro-F1); *θ̂_(b)_*—the metric recomputed on the b-th bootstrap resample of test posts; *Q_2.5_, Q_97.5_*—the 2.5th and 97.5th percentiles of the B bootstrap estimates.

## 4 Results

### 4.1 Experimental setup

All experiments have been performed on the Reddit-based mental health dataset presented above. From 701,783 raw posts, only those that had at least 500 raw words were considered, resulting in 27,690 posts with 5 classes (without taking into account the common *mentalillness* class, as mentioned in Section 5). Posts were divided into 22,152 training instances and 5,538 test instances. For the test set, the topic-blind fixed window baseline created 11,941 chunks (approximately 2.16 per post), while the density-based extractor provided 5,548 chunks (approximately 1.00 per post), as these posts only slightly exceeded the size of the model’s input sequence. Lexicon from the union of the training examples consisted of 354 words. Representations for classical models were generated by TF-IDF with 100,000 features, and the performance was measured using accuracy and macro-averaged F1-score, as defined in Section 3. Dataset statistics are shown in Table 2.

**Table 2:** Dataset statistics for the five-class task (*mentalillness* excluded).

Stage	Count	Notes
Raw posts (full dataset)	701,783	six subreddits
Posts ≥ 500 raw words (5-class)	27,690 (approx.)	long-post filter
Train posts/test posts	22,152/5,538	post-level 80/20 split
Fixed-window chunks (test)	11,941	≈ 2.16 per post
Density regions (test)	5,548	≈ 1.00 per post
Union lexicon size	354 terms	top-75 per class

### 4.2 Effect of data leakage

We start with quantifying the effect of the traditional preprocessing order. Applying chunking and oversampling before the train-test split allows obtaining nearly perfect macro-F1 values for all models, such as 0.96 for XGBoost. In contrast, applying our leakage-safe protocol on the same six-class dataset results in a significant deterioration of all models’ performances, where the most dramatic reduction occurs in the model that initially appeared strongest (Table 3). Such a large deterioration in performance, especially among the highly oversampled classes, suggests that the initial improvement resulted not from true generalization but from data leakage because of the duplicated chunks shared after the split.

**Table 3:** Effect of data leakage on six-class, post-level macro-F1: the naive (leakage-prone) pipeline versus the leakage-safe protocol.

Model	Naive (leakage-prone)	Leakage-safe (ours)
Logistic regression	0.686	0.658
Linear SVM	0.834	0.633
XGBoost	0.960	0.670

This finding repositions the role of the research based on the need for a leakage-resistant evaluation protocol and a scientifically backed approach to extracting texts, rather than the inflated results produced by the conventional pipeline.

### 4.3 Machine learning models

#### 4.3.1 Logistic regression

In the leakage-safe setup, logistic regression obtained a macro-F1 of 0.741 using density extraction, compared with 0.636 for the full-text representation and 0.722 for fixed-window chunking. Since the full-text macro-F1 is outside the 95% confidence interval [0.709, 0.766] of the density approach, there is a statistically significant difference in favor of density extraction as compared to full text for this model. These results indicate that, even for a simple linear classifier, focusing the input on clinically dense passages improves class separability rather than merely reducing input length.

#### 4.3.2 Linear support vector machine

The linear SVM achieved a macro-F1 score of 0.724 using density extraction [0.688, 0.752], compared with 0.745 on the full-text representation and 0.693 on fixed-window chunks. In this case, density extraction is statistically comparable to the full-text baseline, as the full-text score lies within the 95% confidence interval of the density-based result. This suggests that the linear SVM, which effectively exploits high-dimensional sparse TF-IDF representations, is relatively insensitive to input reduction: removing non-informative content neither substantially improves nor degrades performance. However, density extraction still provides a consistent advantage over topic-blind fixed-window segmentation.

#### 4.3.3 XGBoost

XGBoost was the strongest classical model, achieving a macro-F1 score of 0.753 under density extraction [0.725, 0.778], closely matching its 0.762 performance on the full-text representation and exceeding its 0.735 score on fixed-window chunks. As with the linear SVM, this suggests that the tree-based ensemble effectively exploits the full feature space, with density extraction matching—but not surpassing—full-text performance. Importantly, it achieves comparable performance while operating on only a fraction of the input text, indicating that a substantial portion of the remaining content is not essential for classification.

### 4.4 Deep learning models

#### 4.4.1 fastText

fastText shows a clear benefit from density extraction, achieving a macro-F1 score of 0.753 with a 95% confidence interval of [0.724, 0.780], compared with 0.701 for full-text inputs and 0.720 for fixed-window chunking. Since the full-text score lies below the lower bound of the density-based confidence interval, the improvement is statistically significant. Overall, fastText combined with density extraction matches the best-performing classical model while maintaining computational efficiency.

#### 4.4.2 Recurrent neural network (RNN)

The RNN (BiLSTM) achieved a macro-F1 score of 0.637 under density extraction, with a 95% confidence interval of [0.606, 0.667], compared with 0.575 on full-text inputs and 0.621 using fixed-window chunking. The improvement over the full-text baseline is statistically significant. Although the RNN underperforms relative to classical models and fastText, it still benefits from density-based selection, suggesting that concentrating discriminative content reduces sequence length and alleviates the burden of modeling long-range dependencies in recurrent architectures.

#### 4.4.3 TextCNN

The only classifier that suffered from chunking was TextCNN. The macro-F1 score of this algorithm dropped from 0.702 on the full-text dataset to 0.540 when applying fixed window chunking and to 0.530 by using the density-based method (95% CI: 0.502–0.558). Moreover, the performance of TextCNN on minority classes significantly declined and dropped to 0.22 for bipolar disorder and 0.14 for schizophrenia classes. These results should be disclosed since convolutional text classification relies on keeping the contiguous n-gram patterns. The fragmented and non-contiguous chunks of text affect the performance of TextCNN negatively; thus, this method is more appropriate for the analysis of long texts rather than condensed excerpts taken from several parts of the text.

### 4.5 Transformer baseline

The most obvious evidence for this proposed approach can be provided by the context-limited transformer. Given the constraint of the maximum number of tokens that MentalBERT could process at once being equal to 512, the model necessarily needed to discard most of the text from each post. Therefore, the choice of the selected tokens was essential. With naive head truncation, the accuracy of MentalBERT reached 0.848, and the macro-F1 score was 0.767 (95% CI [0.743, 0.791]). In turn, the accuracy increased to 0.866, and the macro-F1 score became 0.790 (95% CI [0.765, 0.812]) under the usage of clinically dense passages as inputs (Table 4). The paired bootstrap test for identical posts demonstrated that the improvement of macro-F1 score (+0.023; 95% CI [+0.006, +0.041], *p* = 0.008) and accuracy (+0.017; 95% CI [+0.009, +0.025], *p* < 0.001) was statistically significant. These results indicate that, when a model is inherently constrained by input length, prioritizing discriminative, clinically dense passages is more effective than retaining only the initial portion of the text.

**Table 4:** MentalBERT (five-class, post-level): naive head-truncation versus density-selected input, with the paired difference.

Input	Accuracy	Macro-F1	95% CI (F1)
Head truncation	0.848	0.767	[0.743, 0.791]
Density selection (ours)	0.866	0.790	[0.765, 0.812]
Paired difference	+0.017***	+0.023**	*p* = 0.008

**p < 0.01, ***p < 0.001 (paired bootstrap, 10,000 resamples).

### 4.6 Comparative analysis

Table 5 highlights the post-level macro-F1 score of all six models based on the three types of input features, while Table 6 presents the per-class F1 scores based on the density-based feature extraction technique. There are two distinct trends that can be observed here. One is that density extraction is superior to the topic-blind fixed-window approach in terms of five of the six models, except TextCNN. The other trend is that density extraction provides statistical improvement over the full text input in terms of logistic regression, RNN, and fastText, whereas the results of SVM and XGBoost are comparable, despite using approximately one extracted region per post.

**Table 5:** Post-level macro-F1 by representation (five-class).

Model	Full text	Fixed window	Density	Density 95% CI
Logistic regression	0.636	0.722	0.741	[0.709, 0.766]
Linear SVM	0.745	0.693	0.724	[0.688, 0.752]
XGBoost	0.762	0.735	0.753	[0.725, 0.778]
fastText	0.701	0.720	0.753	[0.724, 0.780]
RNN (BiLSTM)	0.575	0.621	0.637	[0.606, 0.667]
TextCNN	0.702	0.540	0.530	[0.502, 0.558]

Brackets give the 95% bootstrap CI for the density method.

**Table 6:** Per-class F1 under the density method (five-class, post-level).

Class (*n*)	LR	SVM	XGBoost	RNN	TextCNN
Anxiety (1158)	0.837	0.831	0.835	0.775	0.756
BPD (2909)	0.916	0.920	0.921	0.882	0.872
Bipolar (182)	0.670	0.687	0.693	0.400	0.219
Depression (1222)	0.785	0.784	0.779	0.713	0.669
Schizophrenia (67)	0.495	0.396	0.537	0.413	0.137

Test-set support in parentheses.

The per-class results indicate that performance is highest for well-represented and clearly defined classes (e.g., BPD, up to 0.92, and anxiety, around 0.83), and lowest for underrepresented classes such as bipolar disorder (182 test posts) and schizophrenia (67 test posts), where inter-model differences are more pronounced and where TextCNN exhibits the greatest degradation. These findings are robust to the choice of lexicon size: under the six-class setting with an expanded lexicon (top 200 terms), classical models preserve the same performance ordering, indicating that the observed effects are not sensitive to the specific lexicon cutoff.

## 5 Discussion

We revisited the problem of weighted segmentation for the task of classifying mental health on long Reddit postings, showing that the standard chunk-oversample-split procedure severely overestimates performance due to data leakage. In a leakage-free setting, the apparent superiority of naive weighted chunking largely fades away. To counteract this problem, we introduced the method of domain-informed density extraction, which utilizes a label-agnostic discriminative lexicon extracted from the training set in order to segment informative passages in long texts. Our extensive experiments, along with confidence interval analysis, have shown that domain-informed density extraction reliably outperforms fixed-size window chunking and either achieves equal or better performance compared to full-text representation for all six tested classical and deep learning algorithms, as well as a transformer baseline. This gain is statistically significant for the Logistic Regression, the RNN, and fastText baselines, using much less text input, while density extraction also significantly outperforms naive truncation for the context-constrained MentalBERT algorithm.

The behavior of the TextCNN is what determines the boundary conditions for the presented method. The application of both chunking schemes results in a considerable reduction of the model’s accuracy, especially with regard to the minority classes, due to its dependence on contiguous *n*-grams and disruption thereof. On the contrary, density extraction improves the performance of feature-based models and transformers limited by contextual constraints but may negatively affect models that depend on local contiguity in their inductive biases.

### 5.1 The five-class analysis

Because the *mentalillness* subreddit represents a generic community rather than a specific diagnostic category—evidenced by its diffuse vocabulary and its dispersion across other classes in the confusion matrices, where it achieves only approximately 0.48 F1—we additionally report a secondary five-class analysis excluding this category, alongside the full six-class results, to ensure transparency in the scoping decision. This exclusion is framed as a label-validity correction rather than an attempt to inflate headline performance by removing a difficult class. Importantly, the remaining low-frequency but well-defined classes, such as schizophrenia and bipolar disorder, are retained and reported with their corresponding confidence intervals.

### 5.2 Limitations and future work

A number of constraints affect the conclusions drawn above. While the length of the posts is substantial for social media, they are still only slightly larger than the size of inputs used by the models and tend to place the problem at the beginning, thus restricting the advantage of selecting for context-free models; thus, the density extractor produced one region per post. In addition, subreddit membership is a noisy, self-reported proxy for clinical diagnosis: the data reflect a self-selecting population, and the evaluation is conducted on a single corpus.

A natural next step is to evaluate density extraction on genuinely long, sparse-signal datasets such as the CLEF eRisk collection, where each user is represented by a long posting history and diagnostic signals are distributed across multiple posts. In such settings, the lexicon construction and extraction components remain dataset-agnostic and can be transferred by treating each user history as a single document. Future work may also explore learned selection mechanisms and calibrated, clinically grounded thresholding strategies.

## 6 Conclusion

This paper revisited weighted segmentation for mental health classification of long Reddit posts and showed that the conventional chunk-then-oversample-then-split pipeline inflates performance through data leakage. Under a leakage-safe protocol, the apparent advantage of naive weighted chunking does not persist. We introduce a domain-informed density extraction method that uses a training-derived, label-blind discriminative lexicon to select clinically dense, multi-region passages from long posts. Under rigorous, confidence-interval-backed evaluation across six classical and deep learning models and a transformer baseline, density extraction consistently outperforms fixed-window chunking and matches or exceeds the full-text baseline—significantly for logistic regression, the RNN, and fastText—while using substantially less text. It also significantly outperforms naive truncation for the context-limited MentalBERT.

These findings support leakage-safe evaluation protocols as a default requirement in long-document mental health NLP and position domain-informed density extraction as a practical, model-agnostic representation whose benefits are most pronounced when models are constrained by input length.

## Data Availability

The dataset analyzed in this study is publicly available on Kaggle (https://www.kaggle.com/datasets/kamaruladha/mental-disorders-identification-reddit-nlp; file mental_disorders_reddit.csv).
